# Comparing artificial intelligence- vs clinician-authored summaries of simulated primary care electronic health records

**DOI:** 10.1093/jamiaopen/ooaf082

**Published:** 2025-07-30

**Authors:** Lara Shemtob, Abdullah Nouri, Adam Harvey-Sullivan, Connor S Qiu, Jonathan Martin, Martha Martin, Sara Noden, Tanveer Rob, Ana L Neves, Azeem Majeed, Jonathan Clarke, Thomas Beaney

**Affiliations:** Department of Primary Care and Public Health, Imperial College London, London W12 0BZ, United Kingdom; St Andrews Health Centre, London E3 3FF, United Kingdom; Wolfson Institute of Population Health, Queen Mary University of London, London E1 4NS, United Kingdom; Department of Primary Care and Public Health, Imperial College London, London W12 0BZ, United Kingdom; Department of Primary Care and Population Health, University College London, London NW3 2PF, United Kingdom; Department of Primary Care and Public Health, Imperial College London, London W12 0BZ, United Kingdom; Chiswick Health Practice, London W6 0YD, United Kingdom; General Practitioner, Tower Hamlets, London, United Kingdom; Department of Primary Care and Public Health, Imperial College London, London W12 0BZ, United Kingdom; Department of Primary Care and Public Health, Imperial College London, London W12 0BZ, United Kingdom; Department of Mathematics, Imperial College London, London SW7 2AZ, United Kingdom; The George Institute for Global Health, Imperial College London, London W12 7RZ, United Kingdom

**Keywords:** large language models, generative AI, health informatics, primary care, electronic health records

## Abstract

**Objective:**

To compare clinical summaries generated from simulated patient primary care electronic health records (EHRs) by GPT-4, to summaries generated by clinicians on multiple domains of quality including utility, concision, accuracy, and bias.

**Materials and Methods:**

Seven primary care physicians generated 70 simulated patient EHR notes, each representing 10 patient contacts with the practice over at least 2 years. Each record was summarized by a different clinician and by GPT-4. artificial intelligence (AI)- and clinician-authored summaries were rated blind by clinicians according to 8 domains of quality and an overall rating.

**Results:**

The median time taken for a clinician to read through and assimilate the information in the EHRs before summarizing, was 7 minutes. Clinicians rated clinician-authored summaries higher than AI-authored summaries overall (7.39 vs 7.00 out of 10; *P* = .02), but with greater variability in clinician-authored summary ratings. AI and clinician-authored summaries had similar accuracy and AI-authored summaries were less likely to omit important information and more likely to use patient-friendly language.

**Discussion:**

Although AI-authored summaries were rated slightly lower overall compared with clinician-authored summaries, they demonstrated similar accuracy and greater consistency. This demonstrates potential applications for generating summaries in primary care, particularly given the substantial time taken for clinicians to undertake this work.

**Conclusion:**

The results suggest the feasibility, utility and acceptability of using AI-authored summaries to integrate into EHRs to support clinicians in primary care. AI summarization tools have the potential to improve healthcare productivity, including by enabling clinicians to spend more time on direct patient care.

## Background and significance

Electronic health records (EHRs) contain a huge volume of health-related data. While some data may be entered as “structured” clinical codes, eg, using an alphanumeric code to signify the presence of a diagnosis, this reflects only a small proportion of the information recorded in EHR data in the form of “unstructured,” or “free text” data, which can amount to thousands of words.^1^^,^^2^ Reading this information in preparation for a consultation is a laborious and time-consuming task for clinical staff.^1^ Currently, any summary of patient EHR data must be manually assimilated by a clinician when they need it, eg, before a patient consultation, or when referring a patient to a specialist. Clinicians spend a significant amount of time interacting with EHRs,^3^ and sometimes, more time is spent interacting with EHRs on administrative tasks than is spent delivering direct patient care.^4^ Even when search functions are available to retrieve entries with text relevant to a particular problem, relevant documentation can be missed, leading to safety issues.^5^ Analyses of patient safety reports reveal that difficulty reviewing documentation can contribute to safety events.^6^

Large Language Models (LLMs) have strong language comprehension abilities.^7^^,^^8^ Among them, if LLMs could accurately summarize the extensive information recorded in EHRs, this could improve relevant information retrieval, enhance informational continuity and potentially reduce clinical errors.^9^ Effective summarization could allow for provision of accessible summaries to patients of their own EHR notes. Alternatively, generation of summaries of a patient’s medical history when referring to another healthcare organization could improve continuity between providers. Summaries of a patient’s relevant medical history could also help healthcare professionals to prepare when they review EHR notes before a consultation. To our knowledge, such applications of LLMs are not currently in use but have the potential to reduce clinical workloads while also reducing the risk of vital information being missed.

Existing literature reporting on the application of LLMs within EHRs across healthcare settings largely focuses on the ability of the technology to undertake various information extraction and prediction tasks.^10^ Much of this work has taken place in hospital settings^11^ rather than primary care, where EHRs are arguably richer, including a longitudinal history documenting medical diagnoses, prescriptions and test results across a person’s life.^12^^,^^13^ One study that compared using clinician vs AI suggestions from ChatGPT to optimize clinical decision support in primary care EHRs found those from ChatGPT were evaluated as highly understandable and relevant.^14^ Furthermore, ChatGPT can effectively undertake summarization of clinical dialogues in primary care, as evaluated by human experts.^15^ Other work demonstrates that different LLMs may outperform humans in summarization tasks, including summarizing radiology reports and extracting key information from secondary care notes.^16–21^ To our knowledge, the use of LLMs to summarize free text written data in primary care EHRs has not been investigated. In this study, we compare the quality of EHR summaries relevant to a clinician before consulting with a patient, generated by AI, to those generated by clinicians.

## Materials and methods

### Overview of study design

**Figure 1.**
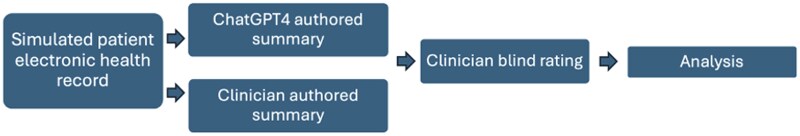
Schematic of study design.

This is a comparative study of the quality of clinical summaries authored by ChatGPT, powered by GPT-4 vs clinicians, with clinicians blinded to the source of the summary during evaluation. We chose this LLM due to its widespread use and existing evidence base in medical informatics research.^22^ GPT-4 was the most advanced version available to us at the time of the study. Figure 1 summarizes the study design. Seven primary care clinicians each generated 10 simulated patient EHR notes representing the 10 most recent longitudinal patient contacts with the practice over at least the last 2 years, resulting in 70 records. These simulated EHRs were generated using templates representative of demographics and chronic condition prevalence in the population of patients registered with general practices in England. Each record was summarized independently by a primary care clinician and by GPT-4. Clinician and GPT-4 summarization took place November 2023-January 2024. AI and clinician-authored summaries were then rated blind by 3 clinicians that had seen neither that record nor summary previously.

### Expert clinician recruitment and training

A convenience sample of 7 doctors working in general practice was recruited via local professional networks, all of whom are co-authors in the project. All clinicians attended 2 90-minute online workshops, which included an introduction to the study and methods. During the workshops, clinicians were given trial EHRs (authored by L.S. and T.B.) to summarize and rate, and had the opportunity to ask questions and achieve consensus on the methods of creating notes and summaries. The first workshop took place before the simulated EHR generation began and focused on how clinicians should approach the task of generating the simulated EHRs and summarization. The second workshop took place before summaries were rated and focused on how clinicians should approach rating the summaries.

### Simulated EHR preparation

To maintain patient confidentiality when using LLMs, no real patient data was used. Instead, a sample of 70 simulated patient EHRs were generated by the clinicians based on profiles designed to use as a template to ensure a representative and varied set of notes. Simulated patient profiles were prepared by L.S., consisting of age, gender, chronic conditions, and repeat medications. These profiles were designed to be representative of demographics according to data on patients registered at G.P. practices in England^23^ and prevalence of health conditions in the population in Quality and Outcomes Framework data (see Appendix S1 for details). Each participant co-author was allocated 10 simulated patient profiles to generate notes in free text form for the 10 most recent GP-entered consultations, reflecting an average patient’s most recent 2-year history of contact with their GP practice.

**Figure 2.**
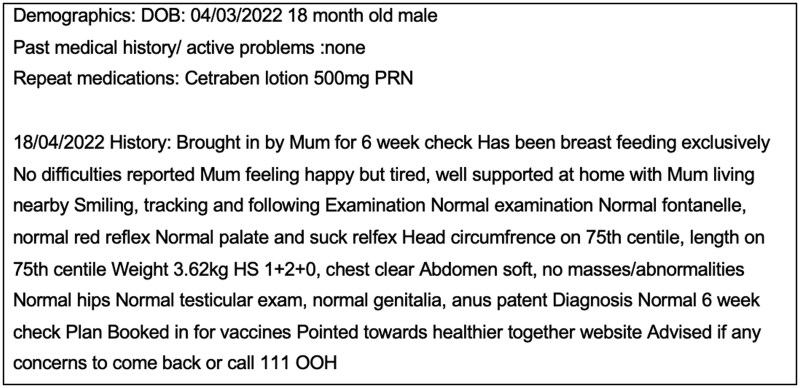
Example template and first (of 10) free text entries for a simulated patient record.

The clinicians attended a first workshop where a consensus approach to generating the EHRs was agreed. Any queries that arose during the writing process were circulated with responses to maintain consistency. All clinicians had current or recent experience of working in general practice, such that both the patient narratives and the writing style would be as reflective of genuine general practice as possible. Documentation styles vary according to clinicians^24^ and having multiple different doctors contribute to note writing helped reflect the range of writing styles in practice. Age, gender, pre-existing conditions and repeat prescriptions were presented as lists at the start of the record, followed by the free text consultation entries. This was reflective of how the author team reviewed structured data in the EHR prior to reviewing free-text data in practice. An example of the start of the record and first free text entry can be seen below in Figure 2. Spelling errors were not corrected to improve the realism. More extensive examples can be seen in Appendix S2.

### GPT-4-authored summaries

ChatGPT powered by GPT-4, used with default settings^25^ was asked to summarize each simulated EHR using the prompt *“This is an example simulated patient record. Please summarise the important points into a paragraph of approximately 100 words intended for a clinician to read before seeing the patient.*” This prompt was followed by the simulated EHR (structured as in Figure 2). Two of the 70 simulated EHRs that were used in the study were also used in a pilot. The 2 EHRs were selected to represent a range of complexity, one set being from a young patient with a less complex history, and another from an older patient with more comorbidities. Before the study, we piloted six different prompts, 3 of which evolved from the best of an initial 3 prompts trialed. The prompt used for the study returned more usable and consistent summaries of those tested (Appendix S2).

### Clinician-authored summaries

Each clinician was assigned at random to summarize 10 EHRs (excluding those EHRs which they generated). As written summarization is not a standard task clinicians undertake in practice, we used the workshops to ensure a consistent approach. Clinicians were provided with an identical briefing to that used for AI: *“This is an example simulated patient record. Please summarise the important points into a paragraph of approximately 100 words intended for a clinician to read before seeing the patient*.” Clinicians were also asked to document the time taken to read through and assimilate the information in the EHR, prior to writing the summary.

### Review of summaries

Each simulated EHR and associated clinician and AI-authored summaries were assigned at random to 3 clinicians (excluding clinicians who had generated the notes or summary). Clinicians were also given access to the full simulated EHR to allow accuracy to be assessed. For each set of EHR notes, the 2 summaries (AI and parallel clinician-authored summary) were independently rated by the 3 clinicians, resulting in each clinician rating 60 summaries in total (30 AI-authored and 30 clinician-authored). Clinicians were not given information on whether the summary was clinician or AI-authored.

**Figure 3.**
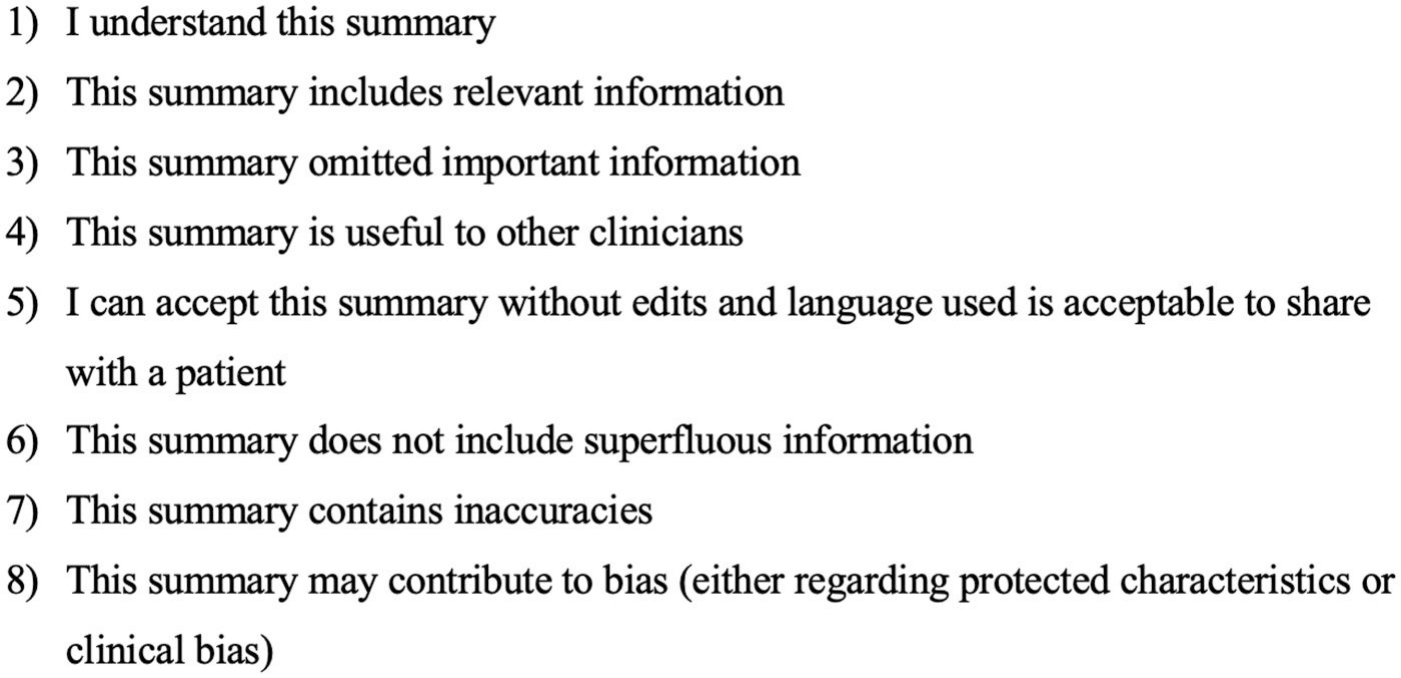
Qualities of summaries rated by clinicians on 5-point Likert scale.

The survey questions were designed to capture multiple domains deemed relevant to a high-quality summary. Each summary was rated on a 5-point Likert scale (1- strongly agree, 2- somewhat agree, 3- neither agree nor disagree, 4- somewhat disagree, 5- strongly disagree) in answer to 8 statements (Figure 3). These were developed from criteria reported in an earlier study, with additional criteria based on input from the study team during the second methodology workshop.^14^

In addition, clinicians were asked to provide an overall rating of each summary from 0 to 10, 10 being the highest score. The question they were asked was “Overall, how would you rate this summary out of 10, 10 being the highest score?” Clinicians were also asked whether they thought the summary was generated by a human or AI. An additional question provided space for free text comments on each summary which were analyzed qualitatively when interrogating reasons for differences in ratings between AI- and clinician-authored summaries for a particular simulated EHR.

### Statistical analysis

Overall ratings for each clinician- and AI-authored summary were compared and we present the minimum, mean and maximum rating for each summary for comparison. A non-parametric Wilcoxon signed rank test was used to assess whether differences in the median ratings between AI- and clinician-authored summaries were statistically significant (*P* < .05). We calculated the pairwise differences for the minimum, mean and maximum overall rating for each clinician- and AI-authored summary to examine the extent of variation of overall rating across clinician-vs AI-authored summaries. In addition, we identified the clinician- and AI-authored summaries whose authorship had been misclassified by the clinicians in the ratings process.

### Ethics

As this project does not use any human participants or their data, ethics approval was not required. All participant clinicians were co-authors on the project.

## Results

A total of 70 patient EHRs were simulated by 7 primary care clinicians. The simulated EHRs are available in full online.^26^ Each EHR was summarized by one clinician and by GPT-4, leading to 140 summaries. Each summary was reviewed by 3 different clinicians, leading to 420 responses (Figure 1). See Appendix S1 for a breakdown of the demographic and chronic condition profiles of the simulated patients.

### Time taken to read and assimilate information contained in simulated EHRs

The time taken to read and assimilate information contained in simulated EHRs was documented for 60 out of 70 clinician-authored summaries (one clinician did not record the time taken). The median time taken was 7 minutes 8 seconds, with a range from 1 minute 25 seconds to 20 minutes and interquartile range of 6 minutes 24 seconds. The median time taken for GPT-4 to summarize information contained in simulated EHRs was 16 seconds with an interquartile range of 9 seconds.

### Overall ratings of summaries

**Figure 4.**
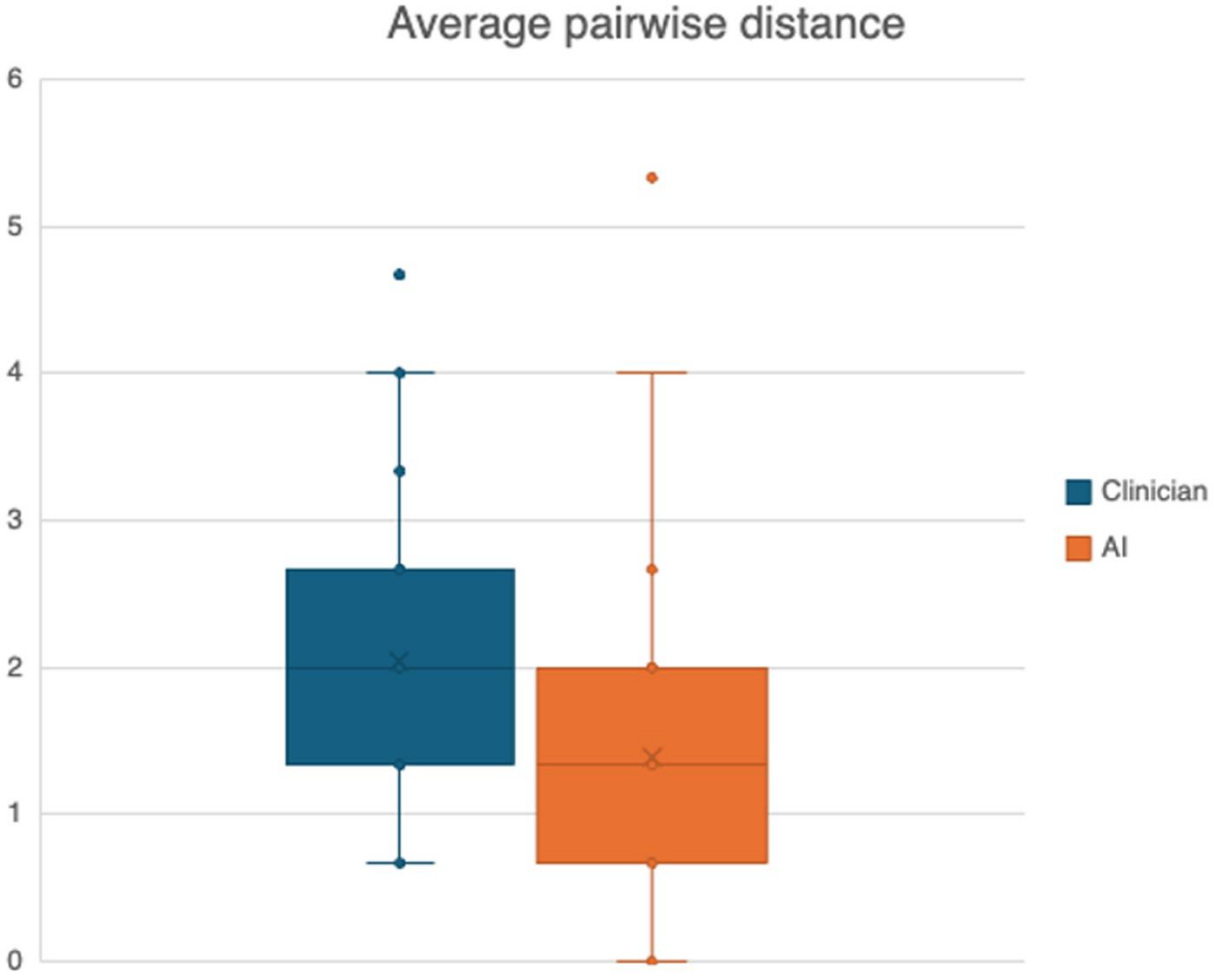
Pairwise distances between the 3 ratings assigned by clinicians for clinician and AI-authored summaries.

Clinicians rated clinician-authored summaries higher than AI-authored summaries (mean score: 7.39 vs 7.00; *P* = .02). There was a larger variability in how clinicians rated clinician-authored summaries [mean pairwise distance (2.04)] than AI-authored summaries [mean pairwise distance (1.38)] Figure 4 and Appendix S3. Clinicians were able to correctly identify the author of the summaries as a clinician or AI 89.8% of the time (Table 1). 16 (7.6%) AI-authored summaries were incorrectly attributed to clinicians and 10 (4.8%) clinician-authored records were incorrectly attributed to AI. Clinicians were unsure of the author of clinician-authored records in 8 cases (3.8%) and of AI-authored records in 9 cases (4.3%).

**Table 1.** Presumed author of summary compared with actual author.

		Clinician assessment			
		Clinician	AI	Unsure	Total
**Actual author**	**Clinician**	192 (91.4%)	10 (4.8%)	8 (3.8%)	210
	**AI**	16 (7.6%)	185 (88.1%)	9 (4.3%)	210

### Quality domains of summaries

**Figure 5.**
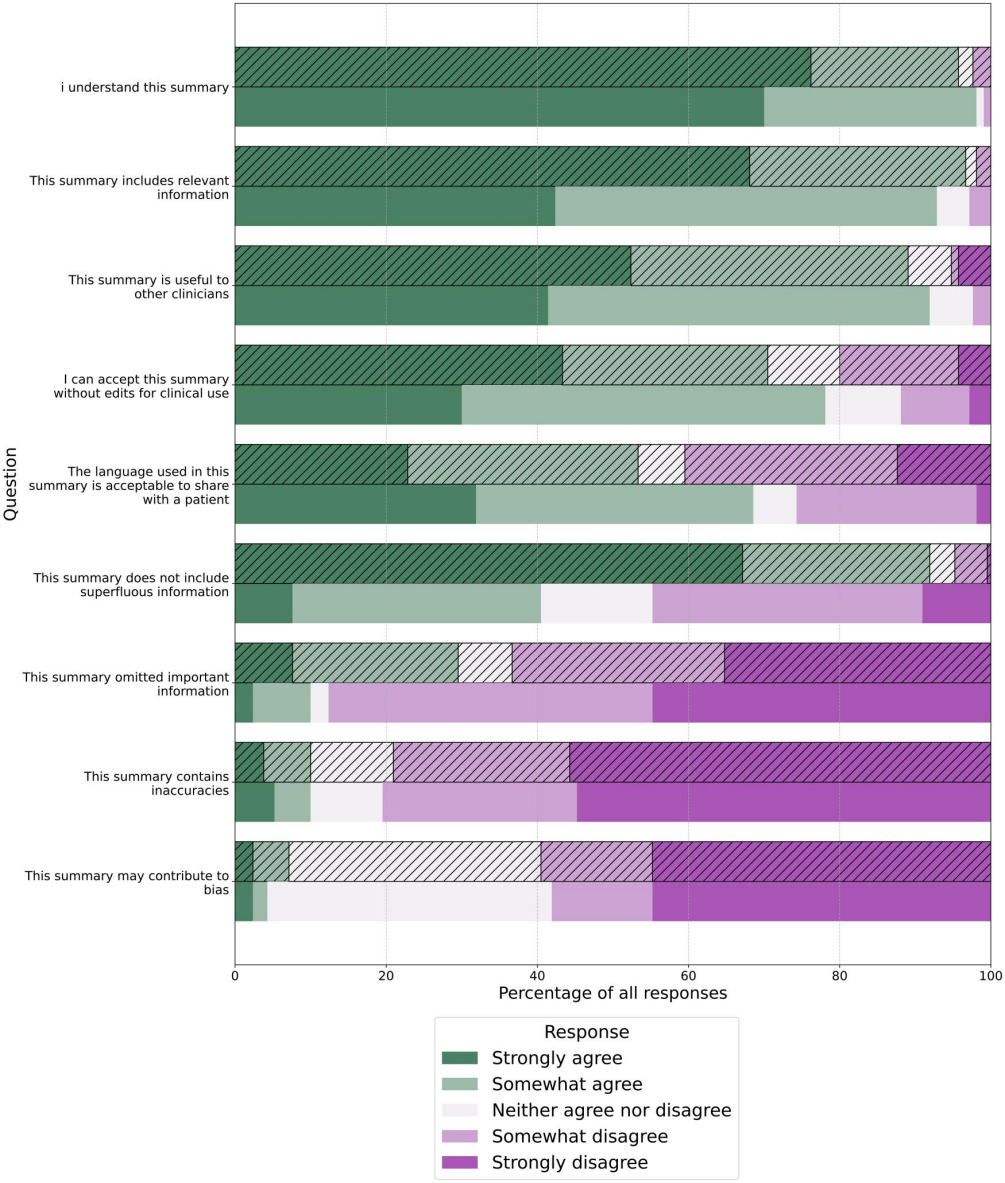
Combined figure of all responses (cross-hatched bars are clinician-authored, plain are AI-authored).

Clinician-authored summaries were rated higher in including relevant information and not including superfluous information (Figure 5). In almost all cases (96.7%), clinicians strongly agreed or somewhat agreed with the statement “This summary includes relevant information” for clinician-authored summaries, compared to 92.9% for AI-authored summaries (Figure 5). AI-authored summaries were perceived to contain more superfluous information. 91.9% of clinicians strongly agreed or somewhat agreed with the statement “This summary does not include superfluous information” for clinician-authored summaries, compared to 40.5% for AI-authored summaries. In contrast, AI-authored summaries were rated higher than clinician-authored summaries in avoiding omission of important information (Figure 5).

Overall, more clinicians strongly agreed or somewhat agreed with the statements “I understand this summary,” “This summary is useful to other clinicians,” and “I can accept this summary without edits for clinical use” for AI-authored than clinician-authored summaries. Inaccuracies and bias were perceived to be rare in both clinician- and AI-authored summaries, with little difference between the 2 (Figure 5). Generally, AI-authored summaries were rated higher than clinician-authored summaries in using language acceptable to share with a patient (Figure 5). 68.6% of clinicians strongly agreed or somewhat agreed with the statement “The language used in this summary is acceptable to share with a patient” for AI-authored summaries compared to 53.3% for clinician-authored summaries (Figure 5).

### Qualitative assessment of summaries

We examined cases where the mean overall rating of the AI-authored summaries was greater than that of the clinician-authored summaries (this happened in 25 out of 70 cases). This revealed consistency in the qualities that AI performed better in compared to clinicians overall, such as not omitting important information and acceptability of language to share with a patient. Generally, clinicians found the AI-authored summaries were clear, concise, chronological, neutral, relevant, accurate and readable while some clinicians found the corresponding clinician-authored summaries lacked detail and context and in one case made a significant omission.

We also interrogated examples where raters identified inaccuracies, incorrect information or hallucination in the summaries (clinician- or AI-authored). This revealed 11 discrete incidences where clinician raters detected hallucination, inaccuracies or incorrect information in AI-authored summaries. Of these, only one was labelled as a hallucination by the rater. In this instance, the AI-authored summary included a “*decision to escalate diabetes medication due to worsening control*,” which the rater accurately identified as hallucination on the basis that on review of the full EHR, “*the dose was increased because the patient tolerated 500mg MR Metformin—the repeat Hba1c had not been done yet.*” There were 12 discrete incidences where clinician raters detected inaccuracies or incorrect information in clinician-authored summaries, and no episodes labelled by raters as hallucination.

## Discussion

Primary care EHRs contain large amounts of patient information which is time-consuming for clinicians to review. This risks important information being missed, potentially compromising patient safety.^5^^,^^6^ Our study demonstrates the feasibility of using a generic LLM to create summaries of patient EHR notes to support clinicians that are overall comparable to the clinician-authored summaries across the domains of quality assessed. The key innovation of this work was creating simulated primary care EHRs, adopting GPT-4 to summarize these, and then designing a strategy to evaluate the quality of these summaries compared to those generated by practicing clinicians.

Summaries authored by AI were overall rated only slightly lower than summaries authored by clinicians (7.00 out of 10, vs 7.39). Clinician-authored summaries contained more relevant information and were less likely to include superfluous information, but AI-authored summaries were easier to understand and more acceptable for use, both by clinicians and patients. Importantly for patient safety, AI-authored summaries were less likely to omit important information than clinician-authored summaries. The median time taken for a clinician to read through the patient record before summarizing, was 7 minutes 8 seconds, which is over 70% of the standard 10-minute appointment time in UK general practice.^27^ This demonstrates the potential of LLM summarization to transform clinical workloads.

Clinician- and AI-authored summaries were rated similarly in accuracy and bias. This provides evidence towards the potential of AI to undertake this task for clinicians in practice. This is important given the concerns with hallucination and bias that have been reported in different contexts, eg, in clinical decision-making tools.^28^^,^^29^ In the context of clinical note summarization, some research suggests hallucination may relate to lack of explicit detail in the notes.^30^ In our study, interrogating the qualitative data submitted by raters revealed a similar incidence of references to inaccuracies or incorrect information in AI-authored vs clinician-authored summaries, and one incident where a rater identified hallucination in an AI-authored summary. Bias is not unique to AI and decision-making by clinical staff may also be biased by factors such as protected characteristics.^31–33^ Ultimately, technology that outperforms humans is the goal. However, in the context of the significant time taken for clinicians to undertake this task, parity in performance may be enough to justify implementation, allowing staff to spend a higher proportion of the consultation on direct patient care.

Our research demonstrates that AI-authored summaries include more patient-friendly language. This is an important finding which suggests AI’s potential use for patient-facing summaries of information in EHRs. Future research could explore which specific features of summaries clinicians and patients value most. Understanding these preferences and reaching consensus on quality criteria will be crucial in optimizing AI tools to complement clinical workflows and ensure their safe and effective integration into healthcare practice.

### Comparison with existing literature

The evidence on the competency of different AI technologies in completing different clinical tasks is rapidly evolving. For example, research demonstrates LLMs can match the level of a specialist clinician in making a diagnosis. Recent research has shown the potential of ChatGPT and other LLMs in clinical summarization tasks, from clinical dialogues to radiology reports, with LLMs performing well and even outperforming clinicians in some contexts^34–36^ Given the lack of objective or gold-standard metrics for assessing the quality of summaries, other studies have adopted the same approach as we used, with expert clinician evaluation of summaries.^15^

Earlier work has found limitations in the ability of LLMs to perform specific tasks relevant to summarization, such as accounting for the chronological ordering of events.^37^ A recent study found an LLM could transform discharge summaries into a format understandable to patients, though not consistently without omissions or inaccuracies.^38^ Heterogeneity of research in both the specific technology and clinical context limits comparability between studies.^39^ To our knowledge, our study is the only published research comparing clinician evaluation of clinician vs AI-authored summaries of simulated primary care EHRs. With AI being increasingly deployed in healthcare workflows, EHR note summarization could be a vanguard use-case for time-saving in healthcare organizations such as the NHS.^40^

### Strengths and limitations

A strength of this study is that 7 active clinicians experienced in primary care generated simulated patient notes, designed to be as representative as possible of a real population in general practice in England. Another strength of the study is that summaries were rated by clinicians who have worked in primary care.

A limitation of the study is that despite the steps taken to make these EHRs as realistic as possible, simulated EHRs may not fully capture the complexity and variability of real patient data, including complex clinical narratives and missing information. This may affect the generalizability of AI models trained on simulated data and their performance in real-world clinical settings. Future work using real patient notes within a secure data environment would enhance the generalizability of findings to real-world health settings.

On average, clinicians took over 7 minutes to read and assimilate the information in the patient notes, which is unlikely to be realized in practice in the context of standard 10-minute GP appointments. Therefore, the quality of summaries authored by clinicians for the purpose of this study may be higher than what is achievable in practice. This could mean that AI-authored summaries would consistently outperform clinician-authored summaries in the context of a live general practice environment. There was more consistency between ratings for AI-authored than clinician-authored summaries which may reflect clinicians curating summaries to their own personal preferences. The finding that AI-authored summaries that perceived as more consistent by clinicians supports the potential of AI to provide a standardized summary for use in clinical practice.

All participating clinicians were authors and convenience-sampled, which introduces potential selection bias. All participating clinicians attended training on summarization for the purposes of standardized methodology in this study, which could minimize the variability of individual summarization methods within the evaluation.

In this study, clinicians were given both the AI and clinician-authored summaries for each set of EHR notes and could accurately discern whether the summary had been generated by a clinician or AI in almost 90% of cases. This is high compared to detection rates reported in other settings.^41^^,^^42^ This may confer bias towards higher scores for clinician summaries if clinicians perceive themselves to be better than AI at summarization and are therefore more likely to award a higher rating. Future research could consider applying blinding approaches using random “style reformatting” to reduce the ability to discern whether summaries are AI- or human-generated.

While clinicians assessed the quality of the summaries for clinical use, the effect of using the summaries in practice is unknown and we need to better understand the clinical significance of differences between AI-generated and clinician-generated summaries. Future research should assess the value of summaries on objective clinical endpoints, such as studying the impact of AI-authored summaries on patient safety errors.

### Implications for practice

We found that a general purpose LLM, ChatGPT, powered by GPT-4, can produce summaries of patient notes which are similarly accurate to those generated by clinicians, with potentially significant time-saving benefits for clinical practice. Such summaries could be read by clinicians to understand a patient’s relevant medical history before a consultation, or to include as a background history alongside a referral to another clinician. LLM summarization could reduce demands on primary care clinicians and in doing so, address risk factors for work-related stress^35^ that are currently contributing to challenges with workforce retention.^36^ An important area for further research is determining the impact of implementing LLM summarization on clinician workload and wellbeing in practice.

However, such technology is not yet ready for implementation. Further research building on ours should aim to determine whether the results demonstrated in this study can be replicated when using real, rather than simulated, patient EHRs. This should be performed within a secure data environment complying with information governance and ethics standards. This should include an evaluation of any systematic algorithmic bias in the use of AI tools.^32–34^ Furthermore, we used a general purpose LLM, but there is scope to improve the performance of the LLM specifically for summarization by fine-tuning a model on high quality examples of summaries, and by refining the prompts used as input to the LLM.

## Conclusions

We demonstrated the use of GPT-4 to generate summaries of free text consultation histories from simulated primary care EHR data, rated slightly lower than clinician-authored summaries overall, but, more consistent, less likely to omit important information and of comparable accuracy to clinician-authored summaries. AI summarization tools could enable clinicians to spend more time on direct patient care, reducing errors resulting from missed information. This study provides proof-of-concept of LLM-based summarization in primary care. Future investigations with more robust blinding, larger and more diverse clinician samples, and genuine (de-identified) patient data will be essential to confirm and extend these findings. Further evaluation to better understand the features that define a high-quality summary, including impact on objective clinical endpoints is also necessary before such technology is implemented in practice. Nevertheless, our findings suggest the feasibility, utility and acceptability of using AI-authored summaries to integrate into EHRs to support clinicians in primary care. By automating tasks, improving clinical decision-making, and enhancing communication, AI has the potential to significantly improve healthcare productivity, leading to better patient outcomes and more efficient healthcare systems.

## Supplementary Material

ooaf082_Supplementary_Data

## Data Availability

The data produced for this study is available on Zenodo.^26^
