# Supplementary material for: Comparing artificial intelligence- vs clinician-authored summaries of simulated primary care electronic health records
Source: JAMIA Open. 2025 Jul 30;8(4):ooaf082. doi: 10.1093/jamiaopen/ooaf082 (PMC12309840; doi:10.1093/jamiaopen/ooaf082)
Supplement: ooaf082_Supplementary_Data [file ooaf082_supplementary_data.zip › Appendix_C.docx]

**Appendix C**

Graphical representation of maximum, minimum and mean overall rating for clinician vs AI summaries
